# Extremely High Preoperative Parathyroid Hormone Associated With Severe Postoperative Hungry Bone Syndrome-Induced Delirium in a Case of Parathyroid Carcinoma in Vietnam: A Case Report

**DOI:** 10.7759/cureus.78213

**Published:** 2025-01-29

**Authors:** Ly Na Dau, Ngoc Diep B Nguyen, Minh D Do, Hoang V Lam

**Affiliations:** 1 Endocrinology and Metabolism, Cho Ray Hospital, Ho Chi Minh, VNM; 2 Pathology, Cho Ray Hospital, Ho Chi Minh, VNM; 3 Endocrinology and Diabetes, Tam Anh Hospital, Ho Chi Minh, VNM

**Keywords:** case report, delirium, hungry bone syndrome, hypocalcemia, parathyroid carcinoma

## Abstract

Parathyroid carcinoma (PC) is one of the rarest cancers, accounting for less than 1% of all cases of primary hyperparathyroidism worldwide. The preoperative level of parathyroid hormone (PTH) has been taken as an essential index in diagnosing and prognosting PC. The conventional range of preoperative PTH was three to 10 times higher than the upper limit. Recently published studies have shown a significantly extensive range of preoperative levels of PTH in different countries, which might change our opinion on probable preoperative PTH levels in PC and its related postoperative complications. We present a case of a 47-year-old Vietnamese male patient suffering from PC with preoperative PTH up to 60 times higher than the upper limit. After the surgery, the patient developed hungry bone syndrome (HBS), manifesting through severe hypocalcemia at 0.04 mmol/l of ionized calcium, which altered his mental status for one week and required a hefty dose of calcium to normalize the patient’s plasma calcium levels. There seems to be no upper limit for PTH levels in PC. A significantly high level of preoperative PTH is an essential warning sign of severe HSB developing postoperatively.

## Introduction

Parathyroid carcinoma (PC) is one of the rarest cancers, accounting for less than 1% of all cases of primary hyperparathyroidism worldwide [1]. In America, PC accounted for 0.005% of all malignancies in the National Cancer Database from 1985 to 1995 [2]. Just more than 3,000 cases have been reported in the English language literature [3].

PC is characterized by severe bone loss and increased plasma calcium levels due to excessive parathyroid hormone (PTH) production from the malignant parathyroid tumor. The key to managing PC is the detection of the carcinoma and its en bloc removal [4].

One of the common postoperative complications of parathyroid surgery is hungry bone syndrome (HBS), which presents with severe hypocalcemia after the surgery and can be fatal without recognition and proper treatment [5]. High preoperative PTH has been considered as one of the risk factors for HBS [6]. The conventional value of preoperative PTH in PCs is around three to 10 times higher than the upper limit, much higher than in parathyroid adenomas. This explains why hypercalcemia tends to be more severe and common, and the clinical picture of osteoporosis-related bone trauma is more dramatic compared to parathyroid adenomas [1]. Recently published studies have shown a significantly wide range of preoperative intact PTH (iPTH) in different countries [7-9].

In this study, we present a case of PC with preoperative iPTH up to 60 times higher than the upper limit. After the surgery, this high level of preoperative iPTH was associated with severe postoperative hypocalcemia of 0.04 mmol/L of ionized calcium, which altered the patient’s mental status for one week and required a massive dose of calcium to normalize plasma calcium levels. Given the rarity of PCs, we hope that our case will contribute to the understanding of the natural course of the condition and will resend the warning message on the importance of the preoperative evaluation and postoperative management of PCs, especially in underprivileged healthcare systems.

## Case presentation

**Figure 1:**
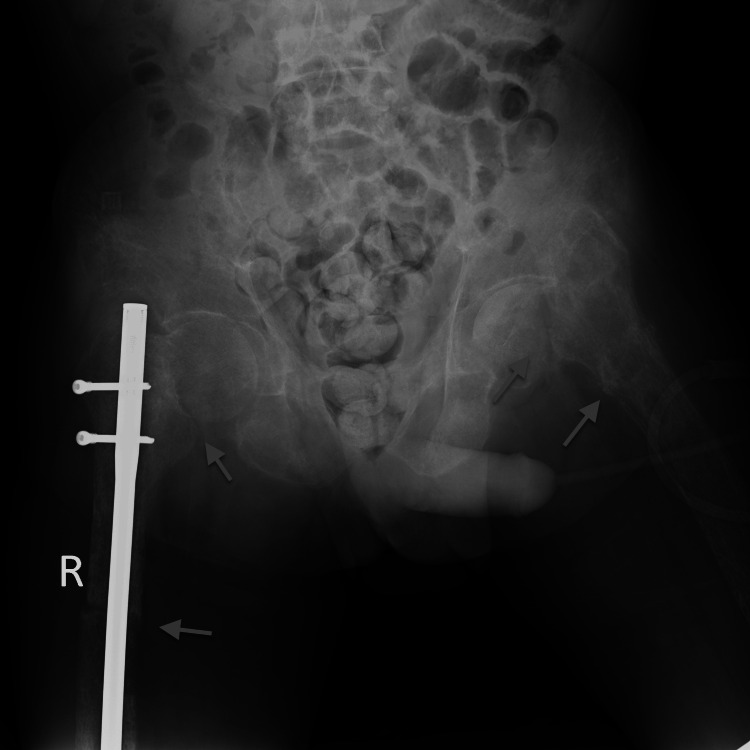
Femoral fractures on both sides

**Figure 2:**
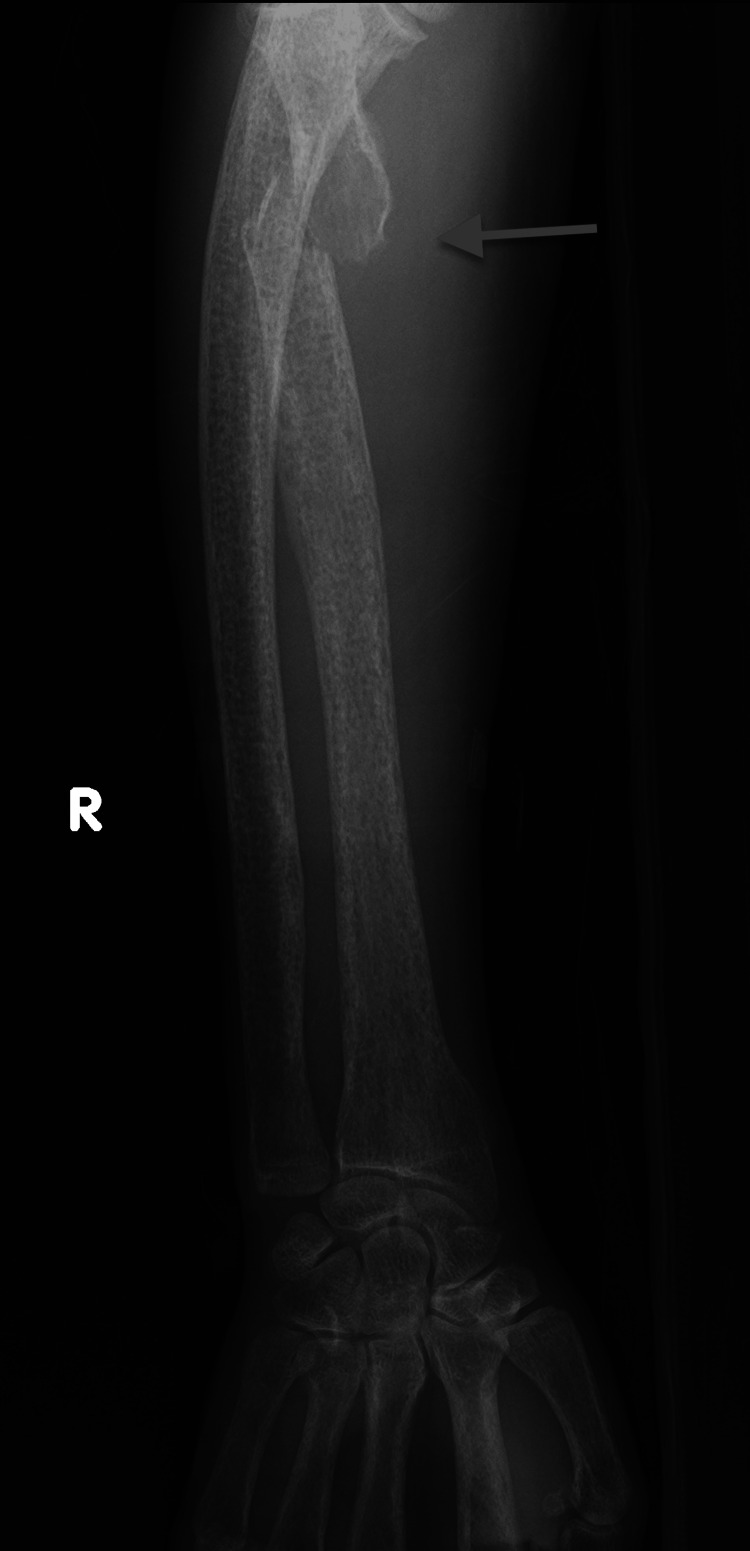
Right radius fracture

A 47-year-old Vietnamese male patient was hospitalized due to newly revealed hypercalcemia. In the prior year, the patient suffered multiple unrelated traumata, including a fracture of the neck of the right femur requiring internal fixation surgery and, a few months later, a fracture of the neck of the left femur and a fracture of the right radius (Figures 1, 2). These fractures were treated at a local hospital.

The patient then attended a nephrology consultation for his newly revealed stage 2 chronic kidney disease. Later, he was diagnosed with primary hyperparathyroidism due to parathyroid adenoma and was hospitalized at the Department of Thoracic Surgery of Cho Ray Hospital. Below are the results of the patient’s tests at Cho Ray Hospital from June 17 to 24, 2020 (Table 1).

**Table 1:** Test results of the patient from June 17 to 24, 2020 iPTH: intact parathyroid hormone, eGFR: estimated glomerular filtration rate

Tests	Results	Normal range
Preoperative iPTH	2335.8	6.5-36.8 pg/ml
Postoperative iPTH	11.3	6.5-36.8 pg/ml
Preoperative total plasma calcium	3.7	2.2-2.6 mmol/l
Postoperative total plasma calcium	2.4	2.2-2.6 mmol/l
Creatinine	1.69	0.7-1.5 mg/dl
eGFR	46.65	>=90 ml/min/1.73 m2

Ultrasound of the neck area showed a lesion behind the left thyroid lobe (likely a parathyroid adenoma) and no thyroid abnormalities. A computed tomography (CT) scan of the neck revealed a 2 cm x 3.5 cm lesion adherent to the left thyroid lobe (possibly a tumor); diffused osteocondensing lesions of the neck and facial bones; focal osteosclerotic lesions (likely malignant) in the inferior maxillary bone and right shoulder bone; and destruction of the cortical bones. A bone density scan showed T-scores of -3.9 at the femoral neck bone and -2.4 for the vertebral bones. Given the aggressiveness of osteoporosis-related bone traumata and the high level of preoperative hypercalcemia and PTH, which is not common in patients with parathyroid adenomas (Table 2), the patient was then diagnosed with suspected PC.

**Table 2:** Comparison of parathyroid carcinomas and parathyroid adenomas

	Parathyroid carcinomas [3]	Parathyroid adenomas [10]
Gender distribution	Equally in males and females	More common in females
Age	44-65	59.2 (range: 22-84)
Most commonly present with	Bone and muscle pain, neuropsychiatric symptoms, kidney stones, hypercalcaemic crisis, osteopenia/osteoporosis, pathological fractures and palpable neck mass	Nephrolithiasis and osteopenia or accidental finding of elevated calcium on laboratory tests for other medical conditions
Preoperative PTH (pg/ml)	566 (range: 31.8-8900)	183.1 (range: 59-1046.8)
Preoperative calcium (mmol/l)	3.39 (range: 2.29-5.98)	2.85 (range: 2.44-3.56)
Ultrasound characters	Infiltration and calcification	Absence of suspicious vascularity, thick capsule, and inhomogeneity

The patient’s surgery was carried out on June 19, 2020. The blood sample collected immediately after surgery showed a significant decrease in iPTH from 2335.8 to 11.3 pg/ml (6.5-36.8 pg/ml), which signaled successful removal of the tumor and a mild decrease in plasma calcium from 3.7 to 3.3 mmol/l. One day later, the patient’s total plasma calcium dropped to 2.4 mmol/L. The patient was discharged five days after surgery with 195 mg of elemental calcium in the form of calcium lactate. Eleven days later, the patient was admitted to a local hospital due to numbness in his fingertips and the tips of his toes. The patient was alert. His plasma ionized calcium was 0.04 mmol/L, which increased to 0.36 after nine days of treatment. No information regarding the dosage of calcium supplements was recorded.

The patient was transferred on July 9, 2020, to the Department of Endocrinology at Cho Ray Hospital because of his fingertips and toes numbness. On admission, the patient was alert, with normal blood pressure. The Chvostek and Trousseau signs were positive. The patient was diagnosed with suspected HBS (Table 3).

**Table 3:** Test results of the patient on July 9, 2020

Tests	Results	Normal range
iPTH	106	6.5-36.8 pg/ml
Ionized calcium	0.49	1-1.5 mmol/l
Albumin	3.5	3.5-5.5 g/dl
Magnesium	0.3	0.7-1.2 mmol/l
Inorganic phosphate	22.1	25-42 mg/l
Creatinine	1.69	0.7-1.5 mg/dl
eGFR	46.65	>= 90 ml/min/1.73 m^2^

The patient then developed an altered mental status. The patient was agitated and speaking nonsense. A head CT scan revealed no abnormalities in brain tissue. The calcium supplement was immediately switched to a combination of intravenous and oral supply; 4,080 mg of elemental calcium was intravenously infused a day. Calcitriol was increased to 1 mcg/day, 1,500 mg elemental calcium daily was given through a nasogastric tube, and 1.5 mg MgSO_4_ was supplied in glucose 5% 500 ml along with CaCl_2_. After four days of intensified treatment, the clinical picture was unchanged. The calcium regimen was up-titrated for a second time, with daily 7,480 mg elemental calcium given intravenously, and calcitriol increased to 1.5 mcg/day. After nine days of the rigorous calcium supplement regimen, the patient became alert. Below are the results of the patient’s tests when he was recovered (Table 4).

**Table 4:** Test results of the patient after recovering from altered mental status iPTH: intact parathyroid hormone, eGFR: estimated glomerular filtration rate

Tests	Results	Normal range
iPTH	62.9	6.5-36.8 pg/ml
Ionized calcium	1.16	1-1.5 mmol/l
Creatinine	1.34	0.7-1.5 mg/dl
eGFR	62.64	>= 90 ml/min/1.73 m^2^
25-OH vitamin D	12.3	25-125 nmol/l

Pathology of the tumor revealed PC (Figures 4-6). The patient was discharged on calcitriol 1.5 mcg/day and 2000 mg/day of elemental calcium orally.

**Figure 3:**
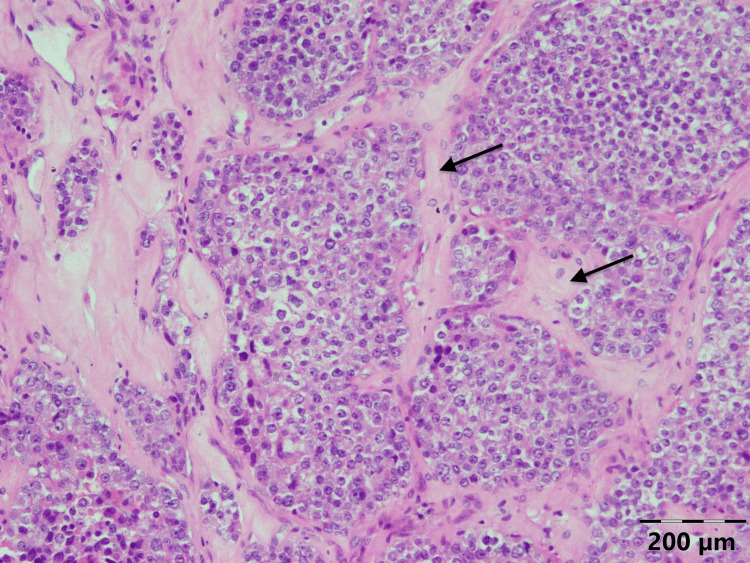
Histology of the parathyroid carcinoma of the patient: broad intratumoral fibrous bands splitting the parenchyma and separating expansile nodules

**Figure 4:**
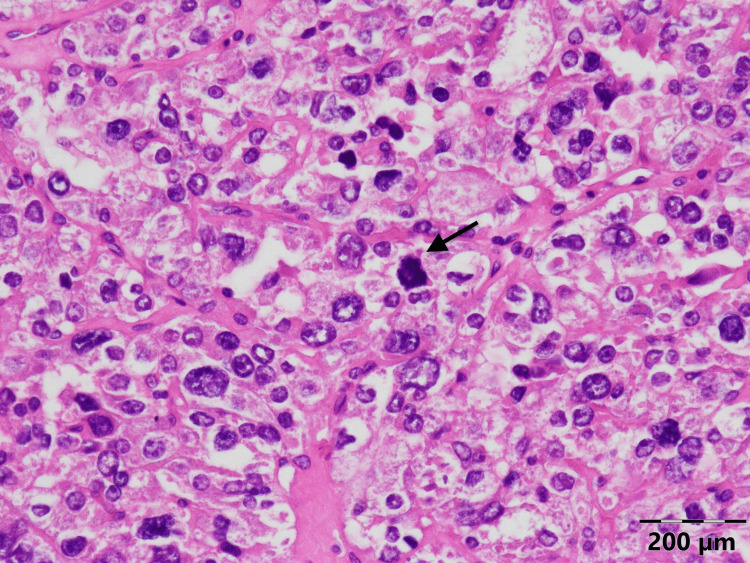
Histology of the parathyroid carcinoma of the patient: mitosis

**Figure 5:**
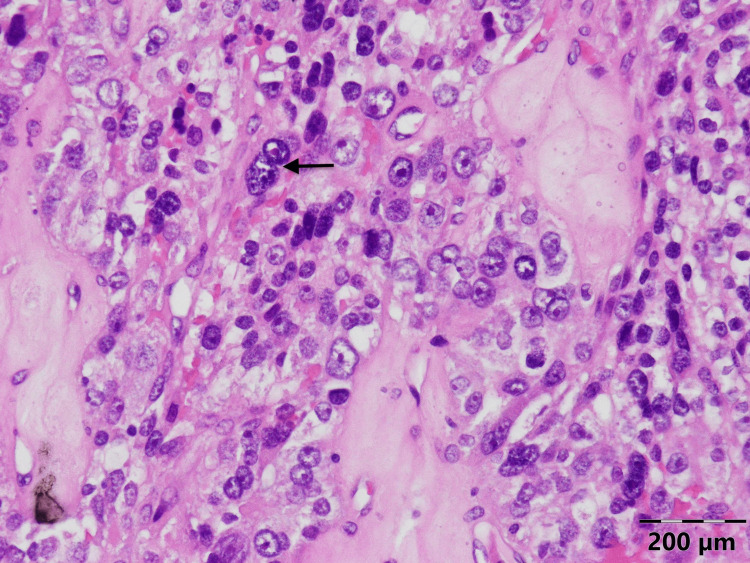
Histology of the parathyroid carcinoma of the patient: cellular atypia and macronuclei in tumor cells

**Figure 6:**
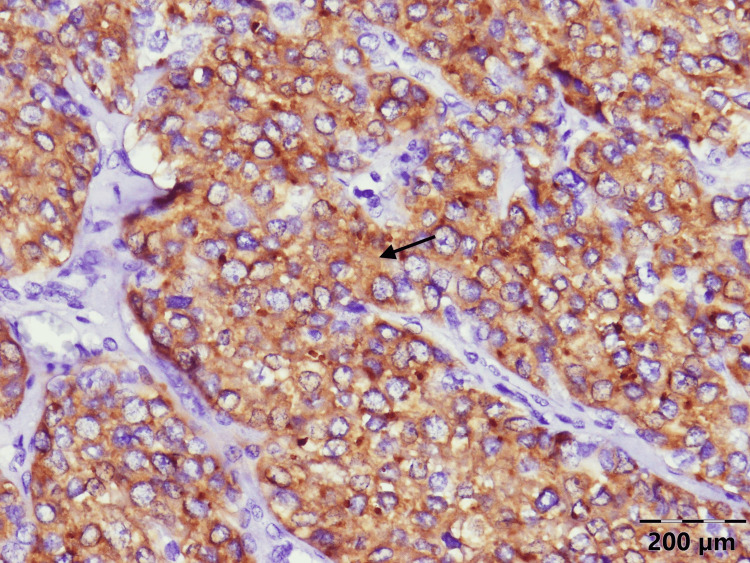
Histology of the parathyroid carcinoma of the patient: positive synaptophysin staining

## Discussion

The patient was diagnosed with HBS secondary to the removal of PC. His level of ionized calcium went down steeply to 0.04 mmol/L. Moreover, he had been suffering from the symptoms of hypocalcemia complicated with delirium.

HBS, defined as profound hypocalcemia (<2.1 mmol/l) appearing after parathyroidectomy and lasting at least four days, can lead to a variety of clinical pictures, from signs of neuromuscular irritability to severe consequences such as general convulsion and congestive heart failure. It can even be fatal if left untreated [5]. HBS is not considered to be true postoperative hypoparathyroidism. HBS is the consequence of the re-regulation of the bone modeling cycle.

Osteoclasts do not have PTH receptors, while there are abundant PTH receptors on osteoblasts, bone lining cells, and osteocytes. Prolonged high PTH exposure stimulates the production of RANKL (receptor activator for nuclear factor Kb ligand) and inhibits the production of OPG (osteoprotegerin) by osteoblasts, bone lining cells, and osteocytes. While RANKL is the main stimulator of osteoclastogenesis, OPG inhibits the differentiation of osteoclasts by binding to RANKL and preventing the binding of RANKL to its original receptor on osteoclasts. Hence, high exposure to PTH stimulates the differentiation of osteoclasts and bone resorption, distorting the normal regulation of the bone remodeling cycle. Bone resorption continues without the commencement of bone formation to complete the remodeling cycle.

Furthermore, long-term high PTH exposure in hyperparathyroidism significantly increases the number of remodeling sites. When this high PTH exposure suddenly stops after the parathyroid removal, osteoblast takes over and initiates the bone formation. Given the prolonged bone resorption phase and remarkably increased number of remodeling sites [11], bone formation will require a massive amount of calcium, resulting in severe hypocalcemia even though PTH concentration might still be in the normal range. Considering the mechanism of HBS, we can assume a close relationship between the severity and longevity of high PTH exposure and the incidence and severity of postoperative HBS.

Long-standing hyperparathyroidism, increased preoperative plasma calcium, PTH, and bone diseases are acknowledged risk factors for developing HBS [12-14]. His severe condition of osteoporosis showed he had been suffering from hyperparathyroidism for a long time before diagnosis. His preoperative iPTH level was 2,335.8 pg/ml, more than 60 times higher than the normal range's upper value. A review by Pia Roser et al., which evaluated 75 studies reporting on more than 3000 patients with PC, has shown a wide range of preoperative PTH levels [3]. The possibly lowest level of preoperative iPTH was 31.8 pg/ml, and the highest level might be 8,900 pg/ml in a report by Lenschow [8]. Roser’s review also included many studies on the Asian population that reported PC cases whose preoperative PTH levels were more than 2,000 pg/ml [9,15,16]. Bae et al. showed the highest preoperative iPTH through two case series on Korean patients was 1,714 pg/ml [17,18]. Through two articles from India, the highest preoperative iPTH was 1,729 pg/ml [10,19], while a study from Japan had their samples’ relatively low average preoperative iPTH with the highest level at 1,230 pg/ml [20]. Even though some articles did not state their reference range of iPTH, we still could see the preoperative iPTH is quite scattered and not racially specific.

Preoperative PTH has been reluctantly considered a risk factor for developing HBS. The results of related studies were conflicting [12,21]. Lee et al. also reported a prevalence of 4.7% of PC among 171 patients with primary hyperparathyroidism diagnosed at their center for 16 years. However, no HBS events after parathyroidectomy were mentioned in this article [18]. The literature on HBS incidence in PC patients is relatively scarce. Perhaps this is due to the rarity of both conditions. Most of the articles are case reports [18,22-25]. Some cases of PC with postoperative HBS had a range of preoperative PTH from 21 to 43 times higher than the upper normal range [22,23]. Scott et al. reported a case of severe HBS after removing a left upper PC that weighed 10 grams. A 35-year-old male patient presented with multiple fractures after minor trauma. Postoperatively, the patient was given intravenous Ca gluconate, equivalent to 20 g of elemental Ca in five days and then oral Ca of 12 g of elemental Ca to stabilize Calcemie [24]. His preoperative PTH was 13.8 ng/ml. However, no normal range was given. Being late in diagnosing might be one of the contributing factors to this extremely high level of iPTH in this patient, a phenomenon often observed in developing countries [26].

The emergence of HBS postoperatively in this patient reminds us of the importance of stratification of patients with hyperparathyroidism before surgery and close observation of plasma calcium after surgery to intensify the treatment appropriately.

## Conclusions

There seems to be no upper limit for PTH levels in PC. The wide range of PTH levels might be related to late diagnosis. A significantly high level of preoperative PTH is an essential warning sign of severe HSB developing postoperatively. This warning sign must be appropriately addressed in the preoperative and postoperative phases.
